# Detecting broad domains and narrow peaks in ChIP-seq data with *hiddenDomains*

**DOI:** 10.1186/s12859-016-0991-z

**Published:** 2016-03-24

**Authors:** Joshua Starmer, Terry Magnuson

**Affiliations:** Department of Genetics, The University of North Carolina at Chapel Hill, Chapel Hill, NC 27599 USA; Lineberger Comprehensive Cancer Center, The University of North Carolina at Chapel Hill, Chapel Hill, NC 27599 USA

**Keywords:** Histone modifications, Computational analysis, ChIP-seq, *hiddenDomains*

## Abstract

**Background:**

Correctly identifying genomic regions enriched with histone modifications and transcription factors is key to understanding their regulatory and developmental roles. Conceptually, these regions are divided into two categories, narrow peaks and broad domains, and different algorithms are used to identify each one. Datasets that span these two categories are often analyzed with a single program for peak calling combined with an ad hoc method for domains.

**Results:**

We developed *hiddenDomains*, which identifies both peaks and domains, and compare it to the leading algorithms using H3K27me3, H3K36me3, GABP, ESR1 and FOXA ChIP-seq datasets. The output from the programs was compared to qPCR-validated enriched and depleted sites, predicted transcription factor binding sites, and highly-transcribed gene bodies. With every method, *hiddenDomains*, performed as well as, if not better than algorithms dedicated to a specific type of analysis.

**Conclusions:**

*hiddenDomains* performs as well as the best domain and peak calling algorithms, making it ideal for analyzing ChIP-seq datasets, especially those that contain a mixture of peaks and domains.

**Electronic supplementary material:**

The online version of this article (doi:10.1186/s12859-016-0991-z) contains supplementary material, which is available to authorized users.

## Background

Histone modifications and DNA binding proteins regulate gene transcription and correctly identifying where they are enriched is crucial to understanding development and cell function [1]. Unbiased genomic surveys of histone modifications and DNA binding proteins can be made using chromatin immunoprecipitation combined with high-throughput sequencing (ChIP-seq). Once sequenced, the reads must be analyzed to identify where they are enriched.

ChIP-seq analysis algorithms have specialized in identifying one of two types of enrichment: broad domains (i.e. histone modifications that cover entire gene bodies) or narrow peaks (i.e. a transcription factor bound to an enhancer). However, the threshold that distinguishes one category from the other is arbitrary and can be spanned by biologically relevant histone modifications. For example, trimethylated H3K27 (H3K27me3), which is correlated with transcriptional repression, can cover entire gene bodies, forming broad domains of enrichment [1], as well as enhancers and transcriptional start sites, forming narrow peaks spanning a small number of nucleosomes [2, 3]. Thus, a full analysis of H3K27me3 can require two separate methods and merging the results in an ad hoc manner [3]. A program that accurately identifies both broad domains and narrow peaks simultaneously would greatly simplify these analyses.

Hidden Markov models (HMMs) are suitable for identifying changes in discrete states, and thus can determine if a region is “enriched” or “depleted” [4]. Importantly, HMMs generate posterior probabilities, providing a measure of confidence that goes beyond the simple binary output of “enriched” or “depleted”. Because it is not always clear where an enriched domain starts and where it ends, posterior probabilities indicate in which parts of the enriched domain users should have high confidence and in which parts they should have only moderate confidence.

We developed *hiddenDomains*, a program that uses an HMM, to identify both enriched peaks and domains simultaneously. It is unique in that it does not need to be tuned to one type of enrichment prior to analysis and does not make assumptions about how reads should be distributed around transcription factor binding sites. We have also added steps to prevent a problem identified in other HMM based enrichment detection programs; inversions in the output, where enriched regions are called depleted. The HMM state optimizations and estimations for transition and Gaussian emission probabilities are performed by the depmixS4 [5] and the HiddenMarkov packages for R. *hiddenDomains* creates BED files that are ready to be displayed in the UCSC genome browser and are colored according to the posterior probabilities, allowing users to select the confidence level they wish to view. We compare *hiddenDomains* to the leading programs designed for broad domains and narrow peaks and show that it performs as well as, if not better, than the best program in each class.

## Results

### Comparing sensitivity and specificity of domain calling

To compare *hiddenDomains* to existing domain detecting methods, we used an H3K27me3 ChIP-seq dataset (GEO: GSE25308) derived from mouse myoblasts that has 145 ChIP-qPCR verified enriched sites and 52 ChIP-qPCR verified depleted sites [6]. The ChIP-seq dataset has 29,694,722 H3K27me3 reads and 39,307,680 reads of sonicated input. The ChIP-qPCR sites allow us to determine sensitivity, the percentage of true positives identified, and specificity, the percentage of true negatives rejected, for each method.

We compared *hiddenDomains* to the following programs for detecting broad domains of ChIP-seq enrichment: *Homer* (version 4.7) [7], *MACS* (version 2.1.0–referred to as *MACSv2* in this manuscript) [8], *PeakRanger* (version 1.18) [9], which includes *BCP* [10] and *CCAT* [11], *Rseg* (version 0.4.8) [12] and *SICER* (version 1.1) [13]. *Homer* and *MACSv2* have options that allow them to specifically search for broad domains of enriched ChIP-seq reads.

**Fig. 1:**
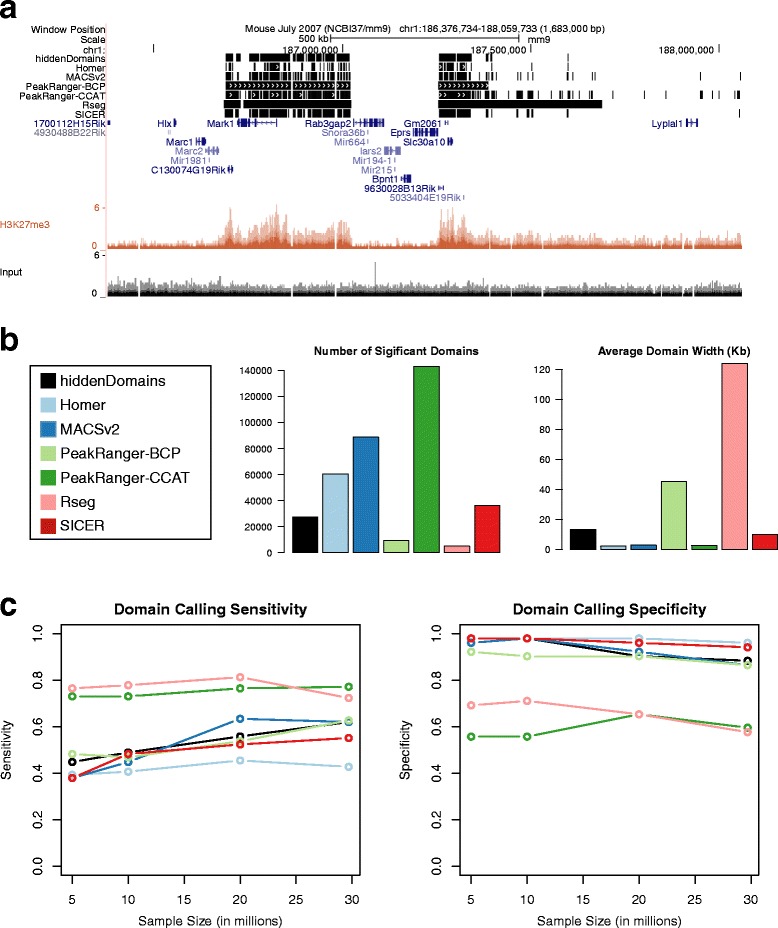
Broad Domains in H3K27me3 ChIP-seq Data. **a** A UCSC Genome Browser screenshot of the ChIP-seq and domains called by the various methods. **b** The number of domains called for each method used and the average domain width. **c** The sensitivity and specificity for the original ChIP-seq dataset and down-sampled versions of it. The *colors* used in the *graphs* represent the same programs listed in the legend for (**b**)

We started by running the domain finding programs on the full dataset and uploaded the output the UCSC genome browser (Fig. 1a). Visual inspection suggested two different domain calling styles; programs either broke enriched domains into smaller fragments and peaks or left larger domains intact. We then quantified the number of domains and their average widths called by all of the programs (Fig. 1b). The programs identified anywhere from 5014 to 143,184 broad domains. In general, the more domains identified, the shorter the average domain. *Rseg* found the fewest and longest domains, averaging 124 Kb per domain. *PeakRanger*-*CCAT* found the most domains, averaging 2.8 Kb per domain. Overall, *Homer*, *MACSv2* and *PeakRanger*-*CCAT* appeared to break enriched domains into smaller fragments and peaks and *hiddenDomains*, *PeakRanger*-*BCP*, *Rseg* and *SICER* left larger domains intact.

We then compared sensitivity and specificity at different read depths, including the full dataset, by down-sampling the H3K27me3 and input reads to 20,000,000, 10,000,000 and 5,000,000 reads (Fig. 1c). The down-sampled datasets were intended to simulate sub-optimal sequencing results. *Rseg*, which found the fewest, but longest domains, and *PeakRanger*-*CCAT*, which found the most, but shortest domains, had the highest sensitivity at all read depths, identifying ~75 % of the ChIP-qPCR verified enriched sites, but also the lowest specificity, failing to reject ~42 % of the ChIP-qPCR verified depleted sites. *hiddenDomains*, *PeakRanger*-*BCP* and *MACSv2* had the next best sensitivities, identifying ~62 % of the verified enriched sites, and only failed to reject 10 % of the verified depleted sites. *SICER* and *HOMER* had the lowest sensitivities, but the highest specificity scores. Among the methods that control the number of false positives in their results with specificities close to 1.0, *hiddenDomains*’s sensitivity is comparable to the best programs in this class.

### Comparing domain widths in an H3K36me3 ChIP-seq dataset

**Fig. 2:**
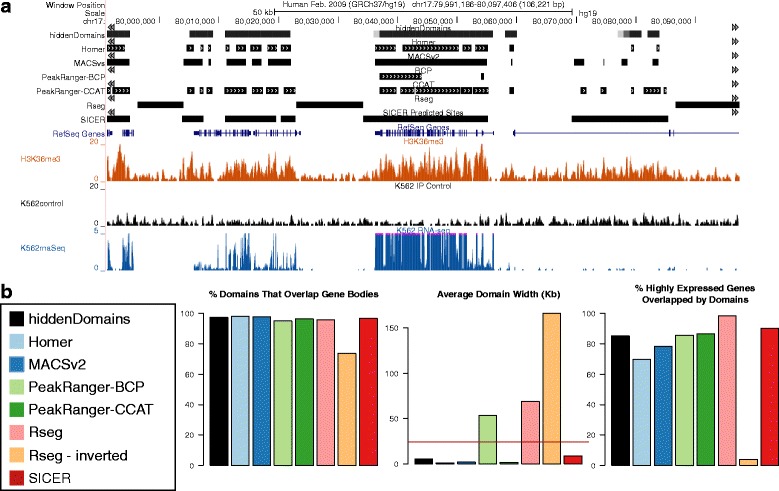
Evaluating Domain Widths with H3K36me3 ChIP-seq Data. **a** A UCSC Genome Browser screenshot of the ChIP-seq and domains called by the various methods. **b** The percentage of domains from each method that overlapped gene bodies, the average domain width (with the average gene body with for transcribed genes indicated with a *red line*) and the percent of highly expressed genes larger than 3 kb that were overlapped by domains called by the various programs

Because there is no gold standard to determine whether a program’s broad domains accurately cover an enriched region, we used the widths of gene bodies in an H3K36me3 ChIP-seq dataset (ENCODE: ENCSR000AKR, ENCFF000BVZ), generated from K562 cells, as an approximation for optimal domain widths. Figure 2a and b shows that all of the programs except for *Rseg* identified domains that overlapped gene bodies almost 100 % of the time. *Rseg*, which also uses HMMs to determine enriched and depleted states, called significantly more domains outside of gene bodies and they often appeared over H3K36me3 depleted regions (Fig. 2a), as if the results had been inverted. To verify that the inverted results were not due to user error, we ran *Rseg* 100 times in a loop, using the same command each time, and observed inverted results in 10 of the runs. Because *Rseg* generated two classes of results, we describe both of them in the remaining analyses, using *Rseg*-*inverted* to refer to the inverted results. Inverting the results is possible with an HMM because defining which state represents enrichment can be arbitrary. In contrast, *hiddenDomains* takes inversion into consideration and adjusts accordingly (see Methods).

H3K36me3 is associated with the chromatin of actively transcribed genes [14]. To identify actively transcribed genes, we paired the ChIP-seq with an RNA-seq dataset (ENCODE: ENCSR000CQL) that had also been performed in K562 cells. After aligning the RNA-seq to the human genome (hg19) with Tophat2 [15], we used DEseq-count [16] to determine the number of reads that mapped to each gene. We then ranked the genes by RPKM and filtered out all genes with RPKM <1 and the average width of the remaining transcribed genes was 24 kb. We then compared the average transcribed gene width to the average domain size called by the various programs (Fig. 2b, middle bar graph, the red line indicates the average transcribed gene width). Both *Rseg* and *Rseg*-*inverted* had the widest domains overall and *Homer*’s were the shortest. *SICER*’s domains were closest in size to the average transcribed genes. *hiddenDomains*’s was a close second, and *PeakRanger*-*BCP* was third. Lastly, we focused on the domains that covered the top 50 highly expressed genes that were longer than 3 Kb (Fig. 2b, the bar chart on the far right). When it did not invert its results, *Rseg* performed best, overlapped 95 % of the gene bodies. *SICER*, *PeakRanger*-*BCP*, *PeakRanger*-*CCAT* and *hiddenDomains* all performed similarly well, calling domains that covered over 80 % of the gene bodies.

### Comparing sensitivity and motif overlap of narrow peak calling

To compare *hiddenDomains* to existing peak calling programs we used a ChIP-seq dataset for GA-binding protein (GABP) (downloaded from http://mendel.stanford.edu/sidowlab/downloads/quest/) in the Jurkat human T lymphoblast cell line [17] that had 150 ChIP-qPCR verified enriched sites [18] defined as having >3-fold enrichment over controls [19]. The ChIP-seq dataset had 7,862,231 GABP reads and 17,404,922 reads of input. Without ChIP-qPCR verified depleted sites we can only calculate sensitivity. In lieu of the ability to calculate specificity, we determined the accuracy of each method as the percentage significant peaks that overlapped 59,618 predicted GABP binding sites identified by *FIMO* [20] with the TRANSFAC GABP motif [21].

We compared *hiddenDomains* to the following programs for detecting narrow peaks of ChIP-seq enrichment: *Homer*, *MACSv2*, *GPS*/*GEM* (version 2.5) [22, 23]. *Homer* and *MACSv2* have options that allow them to specifically search for short peaks of enriched ChIP-seq reads. *SICER*, *PeakRanger*-*BCP* and *PeakRanger*-*CCAT* were excluded from peak detection because their documentation specifically states that they are tuned for domain calling. (The standalone version of *CCAT* can be used for peak detection and is discussed later in the manuscript.) Comparison to additional programs applied to the same transcription factor dataset can be found in [19].

**Fig. 3:**
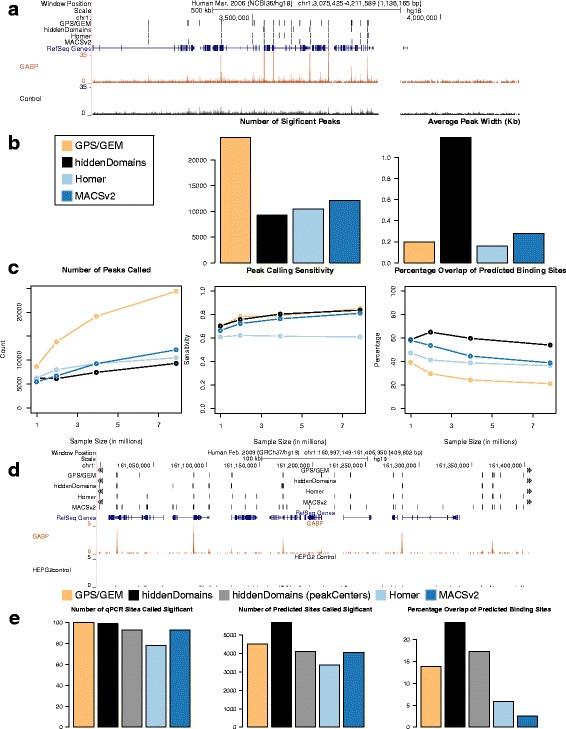
Narrow Peaks in two GABP ChIP-seq Datasets. **a** A UCSC Genome Browser screenshot of the Jurkat ChIP-seq and peaks called by the various methods. **b** The number of peaks called for each method and the average peak width. **c** The number of peaks called, sensitivity and the percentage of peaks that overlapped predicted binding sites for the original ChIP-seq dataset and down-sampled versions of it. The *colors* used in the *graphs* represent the same programs listed in the legend for (**b**). **d** A UCSC Genome Browser screenshot of the ENCODE ChIP-seq and peaks called by the various methods. **e** The number of qPCR validated sites and predicted binding sites overlapped by the peaks and the percentage of peaks that overlapped predicted binding sites called by the various methods

We started by running the programs on the full dataset and uploading the results to the UCSC genome browser (Fig. 3a) and verified that the output from each program overlapped visible peaks. *GPS*/*GEM* identified the most peaks, 24,376, and they were 200 bp wide on average (Fig. 3b). *hiddenDomains* found the smallest number of peaks, 9337, and these were 1.2 kb wide on average, which was to be expected since its default bin size is 1 kb. However, we also tested 212 bp wide bins (212 is the average bin size of the three other peak calling algorithms) and with this setting, *hiddenDomains* identified 11,785 peaks averaging 411 bp wide (Additional file 1: Figure S1).

We then compared the total peaks called, sensitivity, and the percentage of peaks that overlapped predicted GABP binding sites, at different sequencing depths, including the full dataset, by sequentially halving the GABP and input reads to 3,931,116, 1,965,558 and 982,779 reads (Fig. 3c). Again, the down-sampled datasets were intended to simulate sub-optimal sequencing results. At every sample size, *GPS*/*GEM* called the most peaks. In contrast, *hiddenDomains* called the fewest peaks at every sample size larger than the smallest. However, the sensitivity for *GPS*/*GEM* and *hiddenDomains* were almost identical at all sample sizes, indicating that the 2.5-fold increase in the number of peaks called by *GPS*/*GEM* did not provide it with an advantage for identifying ChIP-qPCR verified sites. With the full dataset, GPS/GEM identified 127 (85 %) of the ChIP-qPCR verified loci and *hiddenDomains* identified 125 (84 %). Furthermore, at all sample sizes, *hiddenDomains* had the highest percentage of peaks that overlapped predicted GABP binding sites. With the full dataset, 54 % of the peaks that *hiddenDomains* identified overlapped predicted binding sites, whereas only 21 % of *GPS*/*GEM*’s peaks overlapped predicted binding sites. When *hiddenDomains* used 212 bp wide bins, it maintained the 2nd highest sensitivity (82 %) and the highest percentage overlap of predicted GABP binding sites (40 %) (Additional file 1: Figure S1). Thus, bin size alone cannot account for *hiddenDomains*’s ability to call peaks over predicted GABP binding sites.

Although *GPS*/*GEM*’s sensitivity is 1 % better than *hiddenDomains*’s, it called over 2.5 times as many peaks and only 21 % (5181 of 24,374) of these overlapped predicted GABP binding sites. In contrast, 54 % (5052 of 9337) of *hiddenDomains*’s peaks overlapped predicted GABP binding sites, suggesting that a greater percentage of its results are true-positives. By these metrics, we conclude that *hiddenDomains*’s output was comparable to, if not better than, that from the best peak calling programs.

Because the control dataset for the Jurkat GABP ChIP-seq is relatively noisy compared to newer datasets, we used the peak finding programs on an ENCODE GABP ChIP-seq dataset with much cleaner control data (ENCODE: ENCSR000BJK, ENCSR000BLG) to validate the original results. We used hgLiftOver (http://genome.ucsc.edu/cgi-bin/hgLiftOver) to convert the genomic coordinates for the qPCR validated sites from hg18 to hg19, and we used FIMO and the TRANSFAC GABP motif to predict binding sites in hg19. The new dataset generated results that were very similar to the original, if not more favorable for *hiddenDomains* (Fig. 3d and e). *hiddenDomains* called the fewest significant peaks, but these overlapped almost as many of the qPCR validated binding sites as *MACSv2*, which overlapped the most. The peaks called by *hiddenDomains* overlapped more predicted binding sites than any other method and its percentage of peaks overlapping predicted binding sites was also highest.

Although *hiddenDomains* accurately detects peaks, its dependency on binning reads prevents it from being very precise. To rectify this, we include a helper program, *peakCenters*, which takes the output from *hiddenDomains* and identifies the genomic coordinates for the peak centers. By default, *peakCenters* extends the peak by 100 bp in the up and downstream directions, but this can be changed on the command line. We used *peakCenters* and its default settings on the ENCODE GABPA data and saw that narrower peaks still performed very well, overlapping as many qPCR validated sites as *MACSv2* and more of the predicted binding sites than *MACSv2* and *Homer* (Fig. 3e).

Lastly, *CCAT*, when used separately from the *PeakRanger* suite of programs, can be configured to identify narrow peaks. Using this configuration, we applied it to the ENCODE GABPA dataset. *CCAT* identified 591,231 enriched regions, 18 times more peaks than the next largest number found by *GSP*/*GEM* (32,643). *hiddenDomains*, *Homer* and *MACSv2* all identified close to 10,000 enriched regions, 50 times fewer peaks than *CCAT*’s. *CCAT*’s peaks averaged 1.6Kb wide and covered 968 Mb, over one third of the entire non-N hg19 genome. In comparison, the average number of bases called enriched in the H3K37me3 analysis was only 372 Mb. These results suggest that *CCAT* must have very low specificity and are reminiscent of *CCAT*’s analysis of the H3K27me3 data. With the H3k27me3 data, *CCAT* identified the most domains and had a high sensitivity score, but at the expense of having domains that overlapped many of the validated “depleted” regions and thus, an unacceptably low specificity score (see Fig. 1c). Because of its poor performance with the ENCODE GABP dataset, we excluded *CCAT* from additional narrow peak analyses.

### Analysis of narrow peak calling in additional transcription factor ChIP-seq datatsets

**Fig. 4:**
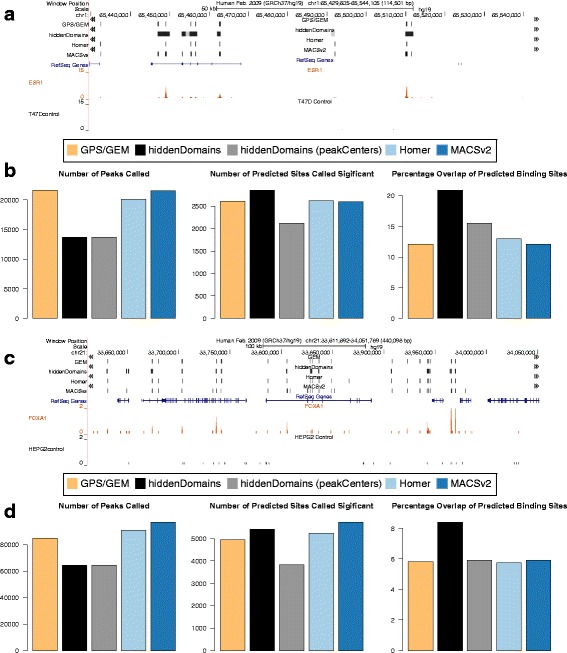
Narrow Peaks in ESR1 and FOXA1 ChIP-seq Data. **a** A UCSC Genome Browser screenshot of the ESR1 ChIP-seq and peaks called by the various methods. **b** The number of peaks called, the number of predicted binding sites overlapped by peaks, and the percentage of peaks that overlapped predicted binding sites for the various methods. **c** A UCSC Genome Browser screenshot of the FOXA1 ChIP-seq and peaks called by the various methods. **d** The number of peaks called, the number of predicted binding sites overlapped by peaks, and the percentage of peaks that overlapped predicted binding sites for the various methods

To further characterize the abilities of the narrow peak calling programs, we applied them to two additional ChIP-seq datasets for the transcription factors Estrogen Receptor 1 (ESR1) (ENCODE: ENCSR000BKN, ENCSR000BMP) and Forkhead Box A1 (FOXA1) (ENCODE: ENCSR000BLE, ENCSR000BLG) (Fig. 4a and c). Just like for GABP, we determined the accuracy of each method as the percentage significant peaks that overlapped the 67,506 predicted ESR1 and the 57,557 predicted FOXA1 binding sites identified by *FIMO* with their respective TRANSFAC motifs.

The results for ESR1 and FOXA1 were similar to the results for GABA1. We found that while *hiddenDomains* found the fewest domains, these overlapped nearly as many predicted binding sites as the other methods (Fig. 4b and d). Furthermore, a much greater percentage of the peaks called by *hiddenDomains* overlapped predicted binding sites for both transcription factors than the other methods (Fig. 4b and d). Lastly, we applied *peakCenters* to *hiddenDomains*’s output for both the ESR1 and FOXA1 datasets and the 200 bp wide peaks had results similar to the GABPA outcomes; there were fewer predicted sites called significant, but the percentage overlap remained the highest of the peak finding programs. These results show that the enriched regions identified by *hiddenDomains* do not overlap predicted binding sites simply because its peaks are wider than the other methods’.

## Discussion

*hiddenDomains* performs exceptionally well using a two state HMM that makes no assumptions about how the reads are distributed around transcription factor binding sites. Although *RSeg* also uses a two state HMM, there are important differences between these two programs. First, *Rseg* uses the difference between two independent negative binomial distributions to model the ChIP and control reads and the HMM parameters are estimated for the entire genome, rather than per chromosome. In contrast, *hiddenDomains* uses a normal distribution to model the difference in normalized read counts in bins with one or more read in either the ChIP or control datasets and the HMM parameters are estimated separately for each chromosome. Second, *RSeg* explicitly incorporates mapability into its model, and *hiddenDomains* implicitly ignores unmappable regions by using samtools to filter out reads that map to them and then ignoring bins with zero reads. Lastly, *Rseg* can invert its output; calling enriched regions depleted and depleted regions enriched. In contrast, *hiddenDomains* evaluates the states after parameter estimation to determine which one represents enrichment. Although these differences in the algorithms are subtle, the differences in the output are dramatic. *Rseg*’s inverted output in the H3K36me3 dataset would lead to erroneous interpretations, and at every sample size in the H3K27me3 dataset *hiddenDomains* had much higher specificity and its domains were much smaller.

In the transcription factor ChIP-seq datasets, we noticed there are several loci that *hiddenDomains* did not call enriched, even though other programs, like *MACSv2* did (Figs. 3a, c and 4c). Many of the sites that *hiddenDomains* excluded from its results had very low ChIP-seq reads that, numerically, were no different from the control dataset. One possibility for why other programs call these sites enriched is their model assumptions (i.e. reads on opposite strands within a specified distance are more likely to indicate transcription factor binding than reads on the same strand). In contrast, *hiddenDomains* does not make assumptions about how reads should be distributed around transcription factor binding sites. Although it might seem like *hiddenDomains*’s simple model would result in excessive false positives, we note that across multiple datasets, the relatively small number of peaks that *hiddenDomains* calls overlap a large number of qPCR validated and predicted binding sites, even when the bin size is set to 212 bp or when *peakCenters* limits them to 200 bp.

Because *hiddenDomains* called relatively few loci significantly enriched, unique loci that were called enriched were rare. However, we did see them from time to time. In one case, this occurred over a very narrow region (less than 100 bp wide) that contained a mound of reads (rather than a vertical rectangle of reads that would suggest a PCR-amplification artifact). This site was called significant because *hiddenDomains* did not make assumptions about how far apart reads should be to indicate enrichment.

*hiddenDomains* is especially useful for data like H3k27me3 ChIP-seq that contain both narrow peaks and broad domains because it does not need to be tuned to either type of enrichment. All other programs that analyze both types of enrichment require setting a parameter that tunes it to one type or the other. However, if knowing the precise location of transcription factor binding site is important, we have three recommendations: 1) using the *peakCenters* program that is included with *hiddenDomains*, 2) using *MACSv2* 3) combining the output from *hiddenDomains* with a specialized binding site program like *GEM*. Furthermore, the default bin size for *hiddenDomains* (1 kb) means that, without changing this parameter, a single peak may span two or more enriched loci (see Fig. 4a). This is acceptable if the user simply wants to know if a transcription factor binds within a region. However, if the user wants higher resolution by default and they know their data only contains narrow peaks, we recommend using *MACSv2*.

## Conclusions

Using ChIP-seq datatsets for H3K27me3, GABP, ESR1 and FOXA1, we have shown that *hiddenDomains*’s sensitivities and specificities are among the best, if not better than, methods that are dedicated to identifying broad domains or narrow peaks. We have also shown that a larger percentage of *hiddenDomains*’s GABP, ESR1 and FOXA1 results overlap predicted binding sites than any other method using the default bin size (1 kb) and much smaller, 212 and 200 bp, bin sizes. Because *hiddenDomains* implements a simple model, and yet fits the data as well, if not better than, more complicated models in a wide variety of situations, we believe it represents a significant improvement over the current state-of-the-art in ChIP-seq analysis.

## Implementation

*hiddenDomains* is a program that consists of three main stages. While more details are given below, briefly, the first stage bins the reads, the second stage creates the HMM and identifies enriched peaks and domains and the third stage converts the results into BED files.

The first step uses *samtools* [24] to filter out reads with low MAPQ scores from a ChIP-seq experiment and counts how many of the remaining reads map to uniformly sized bins spanning the genome of interest. The default minimum MAPQ score, 30, filters out reads that map to multiple locations, have many mismatches, or poorly called base pairs. The default bin size is set to 1 kb, which works well for both broad domains and identifying the presence or absence of transcription factor binding. For more precise peak coordinates for transcription factors, users can set the bin size to 200 bp or smaller, or visually inspect the data in the UCSC Genome Browser for a peak width.

Binned reads, one for a control dataset, if available, and one for a ChIP dataset, are then used as input to the HMM in the second stage. If a control dataset is included, both the ChIP and control datasets are normalized by a factor based on their total read counts. If the smaller of the two datasets has fewer than 10,000,000, 100,000,000 or 1,000,000,000 reads, then the read depths are divided by 1,000,000, 10,000,000 or 100,000,000, respectively. After normalization the new control read counts are subtracted from the new ChIP read counts. This is similar to the method described by Wang, Lunyack and Jordan [25], but not implemented in their program, *BroadPeak*, which does not accept control datasets. Following the normalization step, bins with no reads mapping to them are excluded and *hiddenDomains* truncates the maximum and minimum read counts per bin to minimize the effects that repetitive regions have on estimating variances. If a control dataset is used, the normalized read counts form a normal distribution centered on 0 with a standard deviation that is approximately 5 (Additional file 1: Figure S2). Thus, the default maximum and minimum number of normalized reads in a bin after subtracting the normalized control read counts is 200 and −10, respectively. These values account for the skewing in the data caused by true ChIP-seq enrichments and allow for two times the standard deviation reductions in the depleted areas. Furthermore, these values perform well in practice. However, they can be changed at the users discretion. For example, if *hiddenDomains* only detects repetitive regions, the maximum number of normalized reads can be reduced to 100. *hiddenDomains* then builds and estimates parameters for a two-state HMM, one state modeling enriched regions and one state modeling depleted regions. Parameter estimation is done for each chromosome, using either the depmixS4 [5] or HiddenMarkov packages for R. In practice, we observed that depmixS4 works well with data containing a mixture of broad domains and narrow peaks, and HiddenMarkov works well with data consisting entirely of narrow peaks. *hiddenDomains* tries depmix4 first, and if it fails to converge on parameter estimates, HiddenMarkov is used. On data where both methods succeed, the results are identical (data not shown). With both packages, *hiddenDomains* uses normal distributions to model emission probabilities. The choice of distribution was based on histograms of the normalized binned read counts (Additional file 1: Figure S2). The initial parameters for the “enriched” state are the standard deviation and three times the mean of the normalized bin read counts. The initial parameters for the “depleted” state are the standard deviation and mean of the normalized bin read counts. Formally, the joint likelihood for the series of observations (i.e. the read counts in the bins), *O*_*1:B*_, and latent states, *S*_*1:B*_, given transition and emission parameters, *Θ*, is:

$$ \begin{array}{l}P\left({O}_{1:B},\ {S}_{1:B}\ \Big|\ \varTheta \right) = \hfill \\ {}P\left({S}_1 = i\right)\ P\left({O}_1\ \Big|\ {S}_1 = i\right)\ {\displaystyle {\prod}_{b=1}^{B-1}}P\left({S}_{b+1} = j\ \Big|\ {S}_b = i\right)\ P\left({O}_{b+1}\ \Big|\ {S}_{b+1} = j\right).\hfill \end{array} $$

Where *S*_*b*_ can be either an enriched or depleted state, *P*(*S*_1_ = *i*) is the prior probability of the first bin on a chromosome being in one of those two states, *P*(*O*_1_ | *S*_1_ = *i*) is the probability of emitting the observed read count, *O*_*1*,_ from the initial state, *P*(*S*_*b* + 1_ = *j* | *S*_*b*_ = *i*) is the probability of transitioning from the state assigned to bin *b* to the state assigned to bin *b* + *1*, and lastly, *P*(*O*_*b* + 1_ | *S*_*b* + 1_ = *j*) is the probability of emitting the observed read count at bin *b* + *1* given the state at bin *b* + *1*. Furthermore, *P*(*O*_1_ | *S*_1_ = *i*) and *P*(*O*_*b* + 1_ | *S*_*b* + 1_ = *j*) are normally distributed, with one set of parameters assigned to the enriched state and another set of parameters assigned to the depleted state. In order to obtain maximum likelihood estimates of the model parameters, *Θ*, we first need the marginal likelihood of the observations and this is calculated using the forward algorithm as modified by [26]. This reformulation calculates the gradients of the likelihood at the same time and prevents underflow with both the standard log transformation and using a scaling factor. Thus, the recursive method for calculating *P*(*O*_1 : *B*_| *Θ*) is:

$$ \begin{array}{l}{F}_1\left({O}_1\Big|\ \varTheta \right) = P\left({O}_1,\ {S}_1 = j\right)\hfill \\ {}{F}_b\left({O}_b,\ {S}_b = j\ \Big|\ {O}_{1:\left(b-1\right)}\right) = \left[P\left({O}_b\ \Big|\ {S}_b=j\right){\displaystyle \sum_{i=1}^n}{F}_{b-1}(i)\ P\left({S}_b = j\ \Big|\ {S}_{b-1} = i\right)\right]/{\displaystyle \sum_{i=1}^n}{F}_{b-1}(i)\hfill \end{array} $$

and the log-likelihood is found by taking the log of both sides. Lastly, *Θ* is estimated with the EM algorithm and smoothing parameters are estimated with the forward-backward algorithm.

On rare occasions neither depmixS4 nor HiddenMarkov can converge on parameter estimates for the HMM for a single chromosome when using a control dataset (this did not happen with any of the datasets examined in this manuscript). When this occurs, *hiddenDomains* uses the average parameters estimated from the other chromosomes for the HMM. *hiddenDomains* opts for using parameter estimates per chromosome, rather than always using the average, because using the average results in a slight reduction in sensitivity and specificity (see Additional file 1: Figures S1 and S3). That said, even when the average is used for all chromosomes, *hiddenDomains* continues to rank among the best programs for both broad domain and narrow peak calling.

Once the HMM parameters are estimated, the optimal state, either enriched or depleted, and its posterior probability is assigned to each bin. Because an HMM can arbitrarily assign state 1 or 2 to be the enriched state, *hiddenDomains* identifies the enriched state as the one capturing the highest variance. This information is used as input to the third *hiddenDomains*’s stage, which converts it to a BED file and ensures that the domains’ start and stop coordinates conform to chromosome sizes. When displayed in the UCSC genome browser, each bin is color-coded by its posterior probability. Bins with posterior probabilities greater than 0.9 are black and bins with lower posterior probabilities (>0.8 and >0.7) are sequentially lighter shades of grey.

The *peakCenters* program uses the BEDTools *coverage* tool [27] to count the number of reads that overlap each position in an enriched region. It identifies the peak as the position with the most overlapping reads.

### Software compared to hiddenDomains

We compared *hiddenDomains* to the following programs for detecting broad domains of ChIP-seq enrichment: *Homer* (version 4.7) [7], *MACS* (version 2.1.0–referred to as *MACSv2* in this manuscript) [8], *PeakRanger* (version 1.18) [9], which includes *BCP* [10] and *CCAT* [11], *Rseg* (version 0.4.8) [12] and *SICER* (version 1.1) [13].

We compared *hiddenDomains* to the following programs for detecting narrow peaks of ChIP-seq enrichment: *Homer*, *MACSv2*, *GPS*/*GEM* (version 2.5) [22, 23].

Unless otherwise noted, all examples given in this paper used default settings for all programs. One consistent exception to this was for MACSv2 and Homer, which required specific parameters to identify peaks or domains. For these programs, the appropriate parameters were always set. Default parameters were used because they were, in general, found to be the most stable [28] and these are what most researchers are going to use. See Additional file 1 for example command lines used for each program with each dataset.

## Availability and requirements

Project Name: hiddenDomainsProject Home Page: http://hiddendomains.sourceforge.net/Operating Systems: UNIX, MacOS, WindowsProgramming Languages: Perl and ROther requirements: samtools and, optionally, bedtoolsLicence: GPLv2 All Perl and R scripts are available at SourceForge: https://sourceforge.net/projects/hiddendomains/.

Documentation and a tutorial can be found at: http://hiddendomains.sourceforge.net/.

## Additional file

Additional file 1: This document includes Figures S1, S2 and S3 and example command lines used with each program. (PDF 266 kb)

## Abbreviations

HMM: hidden Markov model
