# Supplementary material for: Detecting broad domains and narrow peaks in ChIP-seq data with hiddenDomains
Source: BMC Bioinformatics. 2016 Mar 24;17:144. doi: 10.1186/s12859-016-0991-z (PMC4806451; doi:10.1186/s12859-016-0991-z)
Supplement: Additional file 1: — This document includes Figures S1, S2 and S3 and example command lines used with each program. (PDF 266 kb) [file 12859_2016_991_MOESM1_ESM.pdf]

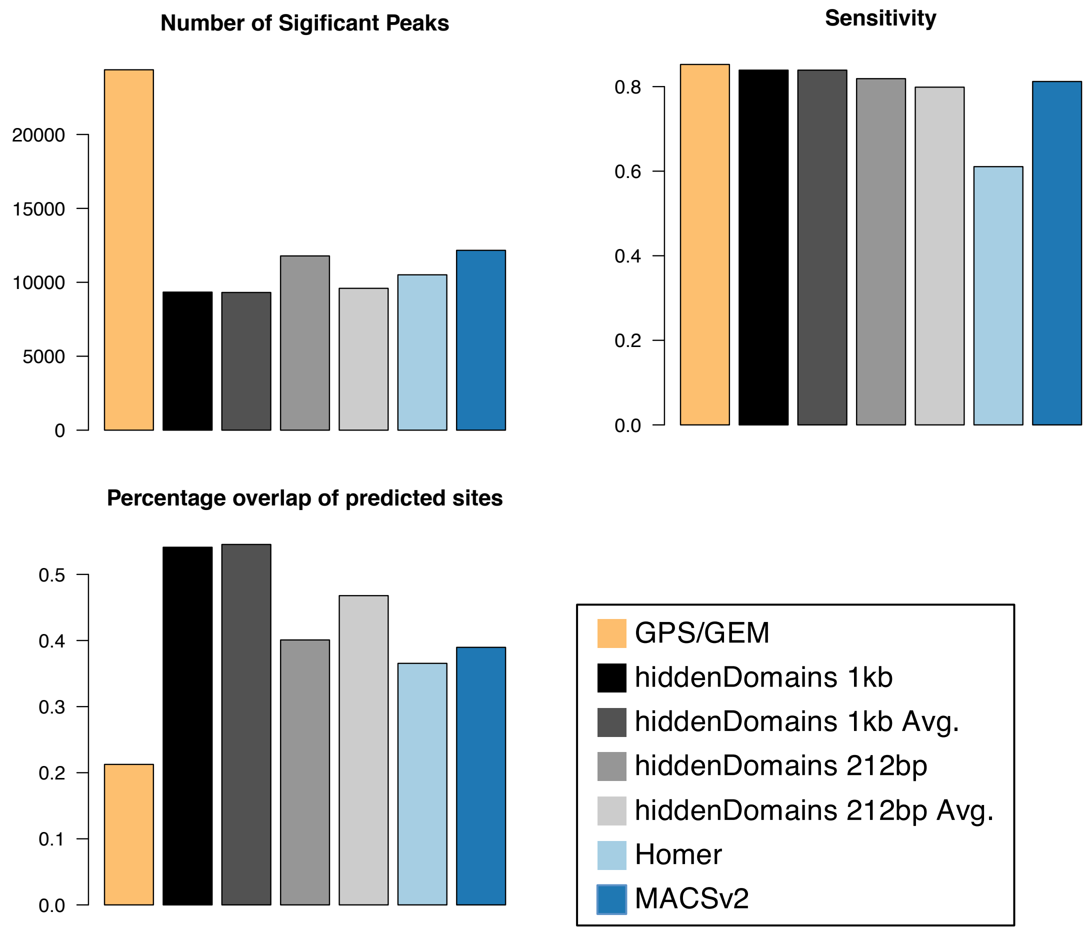

Figure S1: Although the HMM libraries in *hiddenDomains* successfully converged on parameters estimates for all chromosomes in the GABP dataset, we forced it to use the "average" value for both the 1kb and 212bp bin sizes, which is what is used when parameters cannot be estimated. We then calculated the number of significant peaks, sensitivity and the percentage of peaks that overlapped predicted GABP binding sites. Even with the average value used, *hiddenDomains* remains among the best in this class of programs, with high sensitivity and a high percentage of peaks overlapping predicted GABP binding sites.

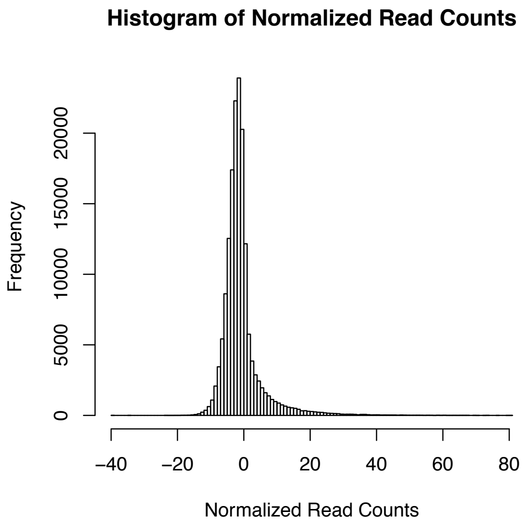

Figure S2: The distribution of reads per bin, after subtracting normalized control reads from normalized ChIP-reads, is approximately normal. The tail on the right side (read counts >0) is larger than the tail on the left side (read counts < 0) indicating that the ChIP sample contains regions of enrichment. Thus, we use one normal distribution for the depleted portions of the genome and one normal distribution for the enriched portions of the genome.

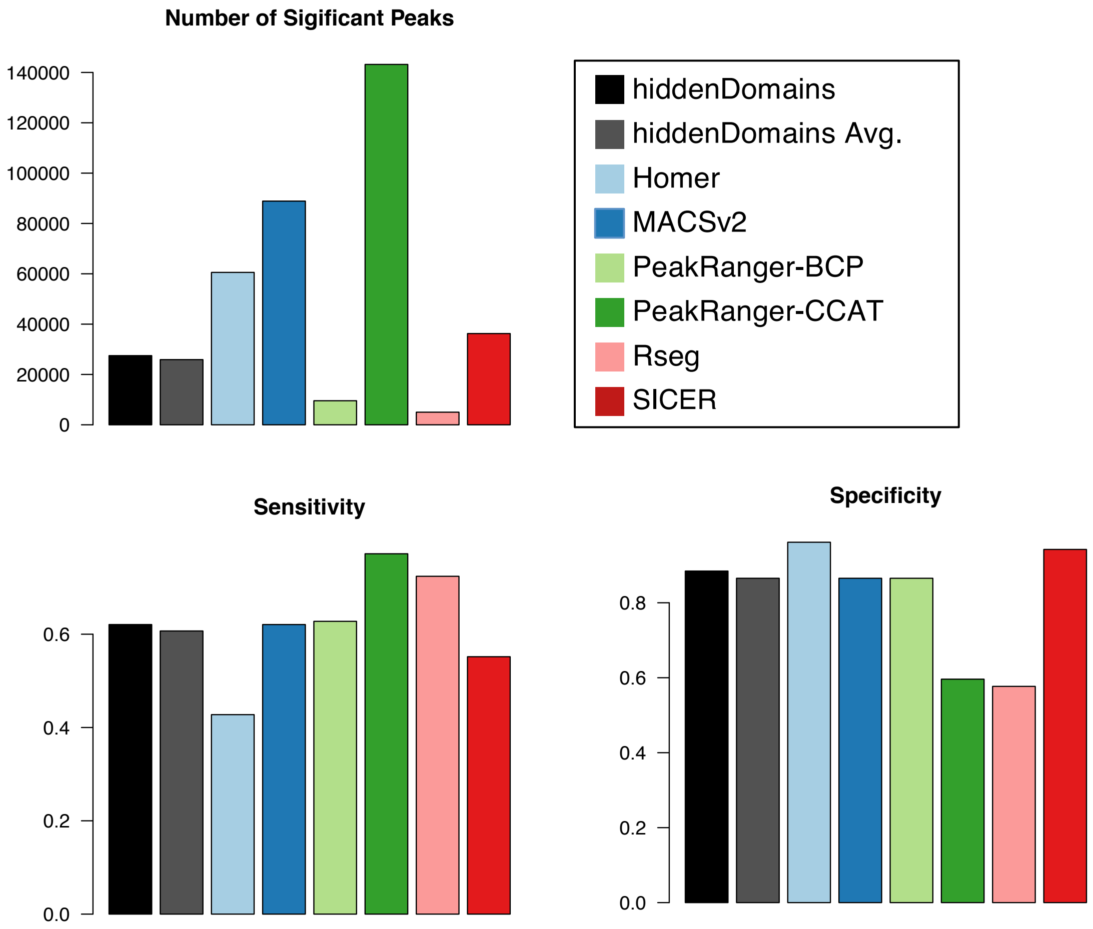

Figure S3: Although the HMM libraries in *hiddenDomains* successfully converged on parameters estimates for all chromosomes in the H3K27me3 dataset, we forced it to use the "average" value, which is what is used when parameters cannot be estimated. We then calculated the number of significant peaks, sensitivity and specificity. Even with the average value used, *hiddenDomains* remains among the best in this class of programs, striking a good compromise between high sensitivity and high specificity.

Example command lines for each program used on the benchmark H3k27me3 and GABPA datasets:

H3k27me3 Benchmark Analysis:

General Comments:

1) The BED and BIN files were generated from the processed reads downloaded from GEO, accession number GSE25308, using a custom perl script.

2) The BED files were sorted with the following Unix commands:

```
sort -k1,1 -k2,2n -k3,3n h3k27me3_mb_bed.txt > h3k27me3_mb_sorted.bed
sort -k1,1 -k2,2n -k3,3n input_sonic_mb_bed.txt > input_sonic_mb_sorted.bed
```

hiddenDomains version 2.0

```
hiddenDomains -b 1000 -c input_sonic_mb_sorted.bed -g ChromInfo_mm9.txt -o hidden_1kb -t
h3k27me3_mb_sorted.bed
```

HPeak, version 2.1

```
perl HPeak.pl -format custom[1,2,6] -t treat_file.txt -n h3k27me3_hpeak -c control_file.txt
```

Where "treat_file.txt" contained a single line with the path to the h3k27me3 BED file and "control_file.txt" contained a single line with the path to the sonicated input BED file.

Rseg, version 0.4.8

After filtering out duplicate reads from the BED file with a custom perl script,

```
bin/rseg-diff -c mouse-mm9-size.bed -out h3k27me3_rseg -i 20 -v -mode 2 -d deadzones-k36-
mm9.bed h3k27me3_mb_sorted_filtered.bed input_mb_sonic_sorted_filtered.bed
```

SICER version 1.1

```
sicer . h3k27me3_mb_sorted.bed input_mb_sonic_sorted.bed . mm9
 4 200 300 0.75 600 .05
```

MACSv2 version 2.1.0.20150731

macs2 callpeak --gsize mm -f BED -t h3k27me3_mb_sorted.bed -c input_mb_sonic_sorted.bed --broad -q 0.05 --name=h3k27me3_macs2

PeakRanger-CCAT version 1.18

peakranger ccat --format bed -d h3k27me3_mb_sorted.bed -c input_mb_sonic_sorted.bed -q 0.05 --output h3k27me3_peakranger_ccat

PeakRanger-BCP version 1.18

peakranger bcp --format bed -d h3k27me3_mb_sorted.bed -c input_mb_sonic_sorted.bed -p 0.05 --output h3k27me3_peakranger_bcp

BroadPeak, downloaded 2015.03.25

After converting the sorted bin files created for *hiddenDomains* to bedGraph format using custom perl scripts:

BroadPeak -i h3k27me3_mb_bins.bedgraph -b 1000 -m h3k27me3_broadpeak -t unsupervised

Homer version 4.7

makeTagDirectory h3k27me3 h3k27me3_mb_sorted.bed -format bed

makeTagDirectory input input_mb_sonic_sorted.bed -format bed

findPeaks h3k27me3/ -style histone -o h3k27me3_homer -i input/

pos2bed.pl h3k27me3_homer > h3k27me3_homer.bed

GABP Benchmark Analysis:

General Comments:

1) The BED and BIN files were generated from the processed reads downloaded from http://mendel.stanford.edu/sidowlab/downloads/quest/ using a custom perl script.

2) The BED files were sorted with the following commands:

sort -k1,1 -k2,2n -k3,3n GABP_hg18.bed > GABP_hg18_sorted.bed
sort -k1,1 -k2,2n -k3,3n control.bed > control_sorted.bed

hiddenDomains version 1.4

hiddenDomains -b 1000 -c control_hg18.bed -g ChromInfo_hg18.txt -o hidden_1kb -t GABP_hg18.bed

GPS/GEM version 2.5

java -Xmx5G -jar gem.jar --d Read_Distribution_default.txt --g ChromInfo_hg18.txt --expt GABP_hg18_sorted.bed --ctrl control_hg18_sorted.bed --out gabp_gem_full --outBED

MACSv2 version 2.1.0.20150731

macs2 callpeak --gsize hs -f BED -t GABP_hg18_sorted.bed -c control_hg18_sorted.bed --name=gabp_macs2

Homer version 4.7

makeTagDirectory gabp_full GABP_hg18_sorted.bed -format bed

makeTagDirectory control_full control_hg18_sorted.bed -format bed

findPeaks gabp_full/ -style factor -o gabp_homer_full -i control_full/

pos2bed.pl gabp_homer_full > gabp_homer_full.bed

CCAT version 3.0

NOTE: CCAT was only run on the ENCODE GABPA dataset (hg19)

bamToBed -i gabp.bam > gabp.bed
bamToBed -i control.bam > control.bed

./CCAT gabp.bed control.bed ChromInfo_hg19.txt ../example/config_TF.txt ccat_gabpa
